# Case Report: A 14-year-old boy with recurrent pancreatitis and autism—response to steroid and rituximab therapy

**DOI:** 10.3389/fped.2025.1594539

**Published:** 2025-09-25

**Authors:** Yu-An Lu, Chieh-Chung Lin, Yen-Chu Huang

**Affiliations:** ^1^ Department of Pathology, Kaohsiung Veterans General Hospital, Kaohsiung, Taiwan; ^2^ Department of Medical Education, Taichung Veterans General Hospital, Taichung, Taiwan; ^3^ Department of Pediatric Gastroenterology, Hepatology, and Nutrition, Children’s Medical Center, Taichung Veterans General Hospital, Taichung, Taiwan; ^4^ Institute of Medicine, College of Medicine, Chung Shan Medical University, Taichung, Taiwan; ^5^ Department of Post-Baccalaureate Medicine, College of Medicine, National Chung Hsing University, Taichung, Taiwan; ^6^ Institute of Biomedical Sciences, National Chung Hsing University, Taichung, Taiwan

**Keywords:** IgG4-related disease, autoimmune pancreatitis, pediatric pancreatitis, pancreatic pseudocyst, autism spectrum disorder

## Abstract

We report the case of a 14-year-old boy with a history of recurrent pancreatitis, autism, and learning disabilities who presented with non-bilious vomiting, epigastric pain, and a progressively enlarging abdominal mass. He was diagnosed with IgG4-related autoimmune pancreatitis. Initial corticosteroid therapy achieved only temporary remission, with disease relapse occurring after 4 months. Rituximab was subsequently introduced, resulting in sustained disease control by depleting B lymphocytes and reducing disease flares, consistent with previous reports. The patient has remained clinically stable for 1 year. This case highlights the clinical presentation, diagnostic challenges, and therapeutic considerations of this rare condition.

## Introduction

IgG4-related disease (IgG4-RD) is a systemic immune-mediated disorder characterized by chronic inflammation and fibrosis involving multiple organs, such as the pancreas, bile ducts, salivary glands, kidneys, and lungs (1). Its hallmark features include elevated serum IgG4 concentrations and marked infiltration of IgG4-positive plasma cells within affected tissues. Autoimmune pancreatitis (AIP) is one of the most prominent manifestations of IgG4-RD and often presents as recurrent pancreatitis, pancreatic masses, or painless obstructive jaundice (2). However, its diagnosis is frequently challenging due to overlapping clinical and radiological features with pancreatic malignancies and other inflammatory conditions.

IgG4-RD is rare in children (3), and its occurrence in children with coexisting neurodevelopmental disorders, such as autism spectrum disorder (ASD), further complicates diagnosis and management. Here, we report a 14-year-old boy with recurrent pancreatitis, autism, and learning disabilities who was ultimately diagnosed with IgG4-RD. Following an initial relapse, he responded well to combination immunosuppressive therapy with corticosteroids and Rituximab, achieving sustained clinical remission.

## Case description

**Figure 1:**
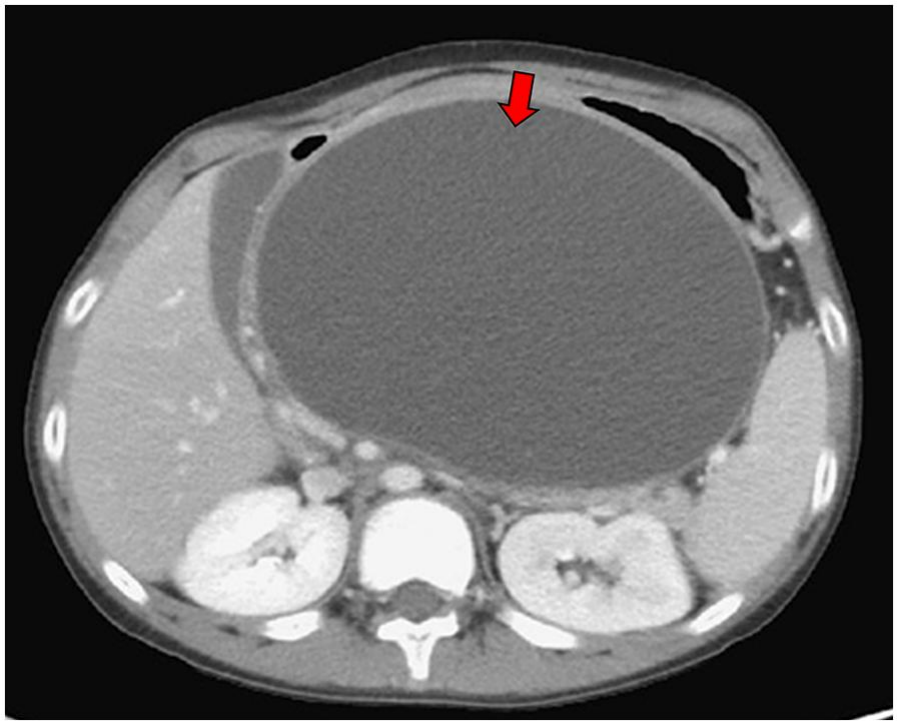
Abdominal computed tomography scan demonstrated pancreatic pseudocyst in transverse view at first visit at our hospital (red arrow).

**Figure 2:**
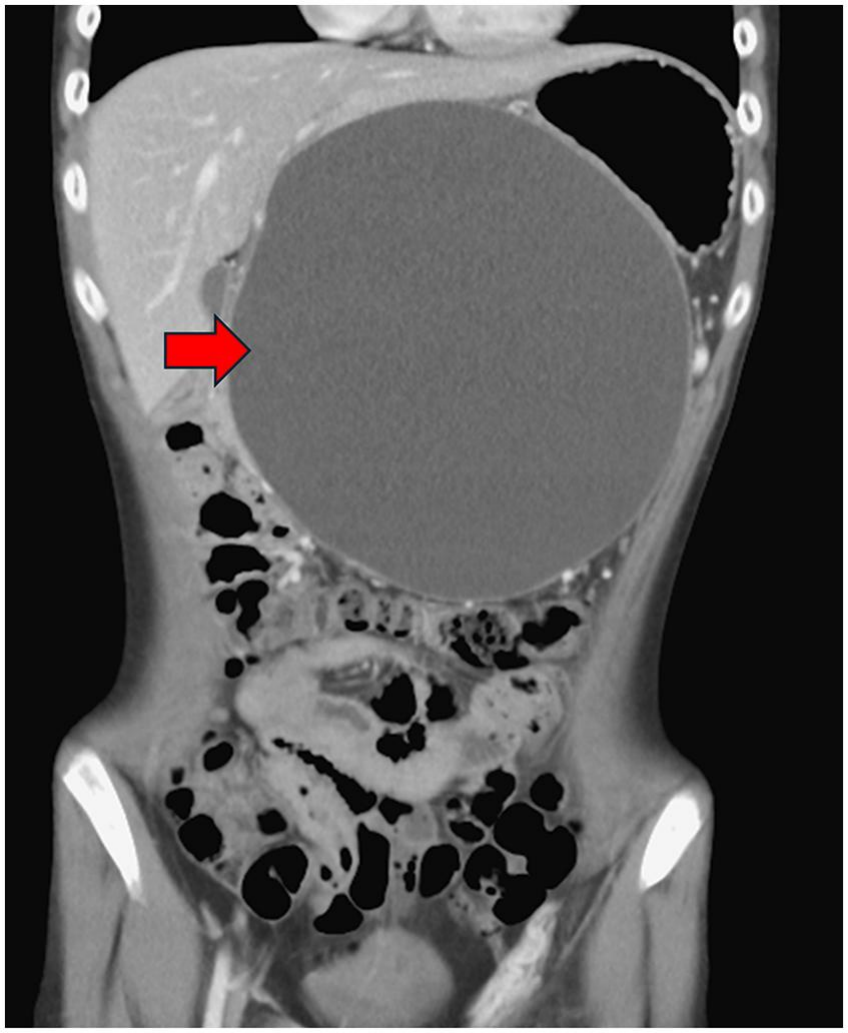
Abdominal computed tomography scan demonstrated pancreatic pseudocyst in coronal view at first visit at our hospital (red arrow).

A 14-year and 6-month-old boy with a history of recurrent pancreatitis, ASD (with communication impairment), and learning disorder presented with frequent non-bilious vomiting for 1 day. Associated symptoms included epigastric fullness, nausea, left-lower quadrant abdominal pain, reduced activity, and decreased oral intake. His mother also reported a progressively enlarging epigastric mass. There was no fever, history of glucose-6-phosphate dehydrogenase deficiency, asthma, allergic rhinitis, or family history of autoimmune disease. On examination, the abdomen was protuberant with an ovoid, non-tender, mass-like lesion measuring approximately 10 cm × 10 cm, without rebound pain. Laboratory findings included serum glucose 107 mg/dL, lipase 155 U/L, and amylase 200 U/L. Venous blood gas analysis showed pH 7.364, HCO_3_ 27.4 and base excess (BE) 1.4, with no electrolyte abnormalities. Abdominal computed tomography scan revealed a large pancreatic pseudocyst measuring approximately 19 cm × 19 cm (Figures 1, 2). Immunologic testing showed elevated total immunoglobulin G (IgG) (2,013 mg/dL), elevated IgG4 (579 mg/dL), positive anti-smooth muscle antibody (1:40), equivocal antinuclear antibody titer (1:80, nuclear fine speckled pattern), and normal complement component 3 (C3), complement component 4 (C4), and transferrin. Antimitochondrial antibody, antiphospholipid antibody, anti-Ro antibody, anti-La protein antibody (targets the Ro and La protein), and anti-neutrophil cytoplasmic antibody were all negative. Endoscopic ultrasound-guided biopsy demonstrated mild fibrosis, acinar atrophy, increased lymphoplasmacytic infiltration, and IgG4-positive plasma cells per high-power field, without obliterative phlebitis.

Based on these findings, a diagnosis of IgG4-positive autoimmune pancreatitis was established. The patient started on oral prednisolone 35 mg daily (0.8 mg/kg/day), tapered gradually according to serial abdominal sonography. During treatment, his body mass index (BMI) remained between 18 and 22, with no obesity. The pseudocyst regressed and eventually resolved with steroid tapering to 5 mg daily (0.1 mg/kg/day). However, 4 months after initial therapy, a recurrent pseudocyst (5.04 cm × 4.56 cm) was detected. Rituximab therapy was initiated, administered as two doses 2 weeks apart, followed by maintenance at 6 months. Subsequently, the pseudocyst regressed and ultimately resolved, with stable clinical conditions maintained to date.

## Discussion

IgG4-RD is a systemic inflammatory and fibrotic condition that can affect virtually any organ. Initially described in 2001 in association with autoimmune pancreatitis, IgG4-RD was later recognized as a systemic disease with characteristic histopathology, including tumor-like swelling, dense IgG4-positive lymphoplasmacytic infiltration, storiform fibrosis, and often—but not always—elevated serum IgG4 levels (4, 5).

Although IgG4-RD may present as isolated organ involvement, multisystem disease is common, affecting the lymph nodes, bile ducts, salivary and lacrimal glands, retroperitoneum, lungs, pleura, and kidneys (6). AIP often mimics pancreatic cancer, as both can present with pancreatic mass lesions and painless jaundice, sometimes with elevated serum IgG4 (7). Other presentations include recurrent acute or chronic pancreatitis, with approximately half of patients developing secondary diabetes. Imaging features often reveal diffuse pancreatic enlargement with a “sausage-shaped” appearance and hypoattenuated halo (8). Treatment options include corticosteroids, immunosuppressants, and B-cell-depleting therapy. Corticosteroids remain the first-line treatment, but relapses are common. Rituximab, an anti-cluster of differentiation 20 (CD20) monoclonal antibody, has emerged as an effective option for steroid-resistant or relapsing disease, inducing remission by depleting B cells and reducing IgG4 production (9, 10).

In pediatric patients with ASD, obesity is sometimes associated with pancreatitis risk. A Japanese study reported obesity in 25% of school-aged children with autism, while other studies suggest a comparable prevalence to the general pediatric population (11). Our patient maintained a normal BMI without antipsychotic use, making obesity-related pancreatitis unlikely.

Children with ASD may present atypically with gastrointestinal or systemic symptoms due to communication difficulties, sometimes manifesting as sleep disturbances or behavioral problems (12). In this case, the patient communicated abdominal pain by pressing his abdomen and asking his mother for analgesics, allowing for a timely medical evaluation.

## Conclusion

This case illustrates the diagnostic and therapeutic challenges of IgG4-RD in pediatric patients with recurrent pancreatitis and coexisting neurodevelopmental disorders. Early recognition of IgG4-related autoimmune pancreatitis is critical to avoid misdiagnosis and inappropriate interventions. While corticosteroids remain the cornerstone of therapy, rituximab provides an effective alternative in relapsing or steroid-refractory cases. Long-term follow-up and further studies are needed to elucidate the disease course and refine management strategies for pediatric IgG4-RD.

## Data Availability

The datasets presented in this article are not readily available because data sharing is not applicable to this article, as no new data were created or analyzed in this study. Requests to access the datasets should be directed to Yen-Chu Huang at doby11915@gmail.com.
